# Associations between Stroke Mortality and Weekend Working by Stroke Specialist Physicians and Registered Nurses: Prospective Multicentre Cohort Study

**DOI:** 10.1371/journal.pmed.1001705

**Published:** 2014-08-19

**Authors:** Benjamin D. Bray, Salma Ayis, James Campbell, Geoffrey C. Cloud, Martin James, Alex Hoffman, Pippa J. Tyrrell, Charles D. A. Wolfe, Anthony G. Rudd

**Affiliations:** 1 Division of Health and Social Care Research, King's College London, London, United Kingdom; 2 Royal College of Physicians, London, United Kingdom; 3 St. George's Hospital, London, United Kingdom; 4 Royal Devon and Exeter NHS Foundation Trust, Devon, United Kingdom; 5 Manchester Academic Health Science Centre, University of Manchester, Salford Royal NHS Foundation Trust, Salford, United Kingdom; 6 National Institute for Health Research Comprehensive Biomedical Research Centre, Guy's and St. Thomas' NHS Foundation Trust, London, United Kingdom; University of Glasgow, United Kingdom

## Abstract

**Background:**

Observational studies have reported higher mortality for patients admitted on weekends. It is not known whether this “weekend effect” is modified by clinical staffing levels on weekends. We aimed to test the hypotheses that rounds by stroke specialist physicians 7 d per week and the ratio of registered nurses to beds on weekends are associated with mortality after stroke.

**Methods and Findings:**

We conducted a prospective cohort study of 103 stroke units (SUs) in England. Data of 56,666 patients with stroke admitted between 1 June 2011 and 1 December 2012 were extracted from a national register of stroke care in England. SU characteristics and staffing levels were derived from cross-sectional survey. Cox proportional hazards models were used to estimate hazard ratios (HRs) of 30-d post-admission mortality, adjusting for case mix, organisational, staffing, and care quality variables. After adjusting for confounders, there was no significant difference in mortality risk for patients admitted to a stroke service with stroke specialist physician rounds fewer than 7 d per week (adjusted HR [aHR] 1.04, 95% CI 0.91–1.18) compared to patients admitted to a service with rounds 7 d per week. There was a dose–response relationship between weekend nurse/bed ratios and mortality risk, with the highest risk of death observed in stroke services with the lowest nurse/bed ratios. In multivariable analysis, patients admitted on a weekend to a SU with 1.5 nurses/ten beds had an estimated adjusted 30-d mortality risk of 15.2% (aHR 1.18, 95% CI 1.07–1.29) compared to 11.2% for patients admitted to a unit with 3.0 nurses/ten beds (aHR 0.85, 95% CI 0.77–0.93), equivalent to one excess death per 25 admissions. The main limitation is the risk of confounding from unmeasured characteristics of stroke services.

**Conclusions:**

Mortality outcomes after stroke are associated with the intensity of weekend staffing by registered nurses but not 7-d/wk ward rounds by stroke specialist physicians. The findings have implications for quality improvement and resource allocation in stroke care.

*Please see later in the article for the Editors' Summary*

## Abstract 2

In a multicenter observational study, Benjamin Bray and colleagues evaluate whether weekend rounds by stroke specialist physicians, or the ratio of registered nurses to beds on weekends, is associated with patient mortality after stroke.

*Please see later in the article for the Editors' Summary*

## Editors' Summary

**Background:**

In a perfect world, a patient admitted to hospital on a weekend or during the night should have as good an outcome as a patient admitted during regular working hours. But several observational studies (investigations that record patient outcomes without intervening in any way; clinical trials, by contrast, test potential healthcare interventions by comparing the outcomes of patients who are deliberately given different treatments) have reported that admission on weekends is associated with a higher mortality (death) rate than admission on weekdays. This “weekend effect” has led to calls for increased medical and nursing staff to be available in hospitals during the weekend and overnight to ensure that the healthcare provided at these times is of equal quality to that provided during regular working hours. In the UK, for example, “seven-day working” has been identified as a policy and service improvement priority for the National Health Service.

**Why Was This Study Done?:**

Few studies have actually tested the relationship between patient outcomes and weekend physician or nurse staffing levels. It could be that patients who are admitted to hospital on the weekend have poor outcomes because they are generally more ill than those admitted on weekdays. Before any health system introduces potentially expensive increases in weekend staffing levels, better evidence that this intervention will improve patient outcomes is needed. In this prospective cohort study (a study that compares the outcomes of groups of people with different baseline characteristics), the researchers ask whether mortality after stroke is associated with weekend working by stroke specialist physicians and registered nurses. Stroke occurs when the brain's blood supply is interrupted by a blood vessel in the brain bursting (hemorrhagic stroke) or being blocked by a blood clot (ischemic stroke). Swift treatment can limit the damage to the brain caused by stroke, but of the 15 million people who have a stroke every year, about 6 million die within a few hours and another 5 million are left disabled.

**What Did the Researchers Do and Find?:**

The researchers extracted clinical data on 56,666 patients who were admitted to stroke units in England over an 18-month period from a national stroke register. They obtained information on the characteristics and staffing levels of the stroke units from a biennial survey of hospitals admitting patients with stroke, and information on deaths among patients with stroke from the national register of deaths. A quarter of the patients were admitted on a weekend, almost half the stroke units provided stroke specialist physician rounds seven days per week, and the remainder provided rounds five days per week. After adjustment for factors that might have affected outcomes (“confounders”) such as stroke severity and the level of acute stroke care available in each stroke unit, there was no significant difference in mortality risk between patients admitted to a stroke unit with rounds seven days/week and patients admitted to a unit with rounds fewer than seven days/week. However, patients admitted on a weekend to a stroke unit with 1.5 nurses/ten beds had a 30-day mortality risk of 15.2%, whereas patients admitted to a unit with 3.0 nurses/ten beds had a mortality risk of 11.2%, a mortality risk difference equivalent to one excess death per 25 admissions.

**What Do These Findings Mean?:**

These findings show that the provision of stroke specialist physician rounds seven days/week in stroke units in England did not influence the (weak) association between weekend admission for stroke and death recorded in this study, but mortality outcomes after stroke were associated with the intensity of weekend staffing by registered nurses. The accuracy of these findings may be affected by the measure used to judge the level of acute care available in each stroke unit and by residual confounding. For example, patients admitted to units with lower nursing levels may have shared other unknown characteristics that increased their risk of dying after stroke. Moreover, this study considered the impact of staffing levels on mortality only and did not consider other relevant outcomes such as long-term disability. Despite these limitations, these findings support the provision of higher weekend ratios of registered nurses to beds in stroke units, but given the high costs of increasing weekend staffing levels, it is important that controlled trials of different models of physician and nursing staffing are undertaken as soon as possible.

**Additional Information:**

Please access these websites via the online version of this summary at http://dx.doi.org/10.1371/journal.pmed.1001705.

- This study is further discussed in a *PLOS Medicine* Perspective by Meeta Kerlin
- Information about plans to introduce seven-day working into the National Health Service in England is available; the 2013 publication “NHS Services—Open Seven Days a Week: Every Day Counts” provides examples of how hospitals across England are working together to provide routine healthcare services seven days a week; a “Behind the Headlines” article on the UK National Health Service Choices website describes a recent observational study that investigated the association between admission to hospital on the weekend and death, and newspaper coverage of the study's results; the Choices website also provides information about stroke for patients and their families, including personal stories
- A US nurses' site includes information on the association of nurse staffing with patient safety
- The US National Institute of Neurological Disorders and Stroke provides information about all aspects of stroke (in English and Spanish); its Know Stroke site provides educational materials about stroke prevention, treatment, and rehabilitation, including personal stories (in English and Spanish); the US National Institute of Health SeniorHealth website has additional information about stroke
- The Internet Stroke Center provides detailed information about stroke for patients, families, and health professionals (in English and Spanish)

## Introduction

Providing healthcare on weekends and overnight that is of equal quality to the care provided in regular working hours is a major challenge to healthcare systems. In the United Kingdom, the need for “seven-day working” has been identified as a policy and service improvement priority for the National Health Service [1]. The move towards 7-d/wk services has been partly driven by observational data demonstrating an association between weekend admission and worse patient outcomes in a number of countries and health systems [2]–[7]. However, not all studies have found an association between weekend admission and poor outcomes [8],[9]. It is unclear whether these findings are the result of confounding from illness severity or they reflect differences in the nature and quality of healthcare services on weekends. Moreover, the question of whether the organisation of healthcare services on weekends affects patient outcomes, and if so, how, has received very little research attention. In particular, there is a lack of studies testing the relationship between patient outcomes and the level of physician or nurse staffing on weekends. Calls to increase the intensity of medical and nursing staffing on weekends are therefore largely based on the expectation, rather than evidence, that potentially expensive changes in staffing will result in improvements in quality.

We therefore aimed to describe the relationship between stroke mortality and weekend staffing intensity by stroke specialist physicians and registered nurses in a large multicentre sample of stroke services in England. Stroke is common, a major cause of mortality [10], and results in a large burden on individuals and society [11],[12]. The study was designed to test whether observational data were consistent with the hypotheses that being admitted to a stroke unit (SU) without 7-d/wk ward rounds by stroke specialist physicians or with lower ratios of registered nurses to SU beds resulted in higher mortality in patients with stroke.

## Methods

### Ethics

Ethical approval of the Stroke Improvement National Audit Programme (SINAP) was granted by the Ethics and Confidentiality Committee of the National Information Governance Board for Health and Social Care. Further ethical approval was not sought.

### Data Sources

Data were drawn from two datasets of stroke care in England—one of the organisational characteristics of SUs and one of stroke patient characteristics and process of care. Patient-level data were extracted from SINAP, a prospective register of stroke patients admitted to participating hospitals in England (66% of eligible hospitals in England) [13]. Hospitals were not reimbursed for participation and were encouraged to provide data on all patients with ischaemic stroke or primary intracerebral haemorrhage. Patients with subarachnoid haemorrhage or transient ischaemic attack were not included. Ischaemic stroke was subtyped according to the Oxfordshire Community Stroke Project (OCSP) classification, using clinical characteristics [14]. The SINAP dataset includes patient characteristics, stroke phenotype, and details of the first 72 h of care. Data were entered prospectively by the patient's clinical team via a secure online portal, with real-time data validation checks. Mortality status was obtained by linkage to the English national register of deaths [15]. Case ascertainment per hospital was estimated by comparing against administrative data returns (the Health & Social Care Information Centre's Hospital Episode Statistics). Data linkage was carried out via a secure third party [16], and the investigators were provided with an anonymised dataset after removal of patient identifiers. All adult patients (aged ≥18 y) admitted with stroke from 1 June 2011 to 1 December 2012 were included in the analysis.

Data on the characteristics of SUs, including medical, registered nursing, and unregistered healthcare assistant staffing levels, were obtained through the Sentinel Stroke National Audit Programme Acute Organisational Audit [17]. This is a biennial survey of all hospitals in England, Wales, and Northern Ireland admitting patients with stroke, benchmarking the organisational characteristics of stroke services against national standards and guidelines. The data were collected in July 2012 and represent a self-reported snapshot of SU organisational characteristics at that time, including staffing levels and bed numbers. There is no stroke centre certification scheme in the UK, and so we used the presence of 24/7 on-site thrombolysis provision as a marker of stroke centres providing more comprehensive acute stroke care. In the survey, SUs reported the number of registered nurses usually working at 10 a.m. on weekdays, Saturdays, and Sundays/public holidays. The numbers for Saturdays and Sundays/public holidays were averaged to give an estimate for the weekend. Ratios were calculated as the number of registered nurses working per ten SU beds, including only beds to which patients with acute stroke are typically admitted and excluding beds used solely for stroke rehabilitation or post-72-h care.

### Analysis

We explored the association between weekend admission and two measures of weekend staffing: first, the presence or not of 7-d/wk stroke specialist physician ward rounds, and second, the ratio of registered nurses working on the weekend per ten SU beds. Patients were stratified according to day of admission (weekday or weekend) and by the provision of weekend medical and nurse staffing in the first SU to which they were admitted after stroke. Stroke services were categorized as having rounds 7 d/wk or fewer than 7 d/wk, and more detail on the SUs in these two groups is provided in Tables 1 and 2. Nursing ratios were included as a continuous variable, using flexible model fitting approaches that allowed for non-linear associations between nursing ratios and mortality. A secondary analysis categorised the weekend nurse staffing into four groups: <1.5, 1.5–1.9, 2.0–2.9, and ≥3.0 nurses per ten beds. There were very few missing data, and models were fitted to 56,211 patients, 99% of the available sample.

**Table 1:** Characteristics of patient population, organisational characteristics of stroke units, process measures of care quality, and crude mortality rate by presence of physician ward rounds 7 d/wk versus <7 d/wk.

Category	Characteristic	Stroke Specialist Physician Rounds		*p*-Value
		7 d/wk	<7 d/wk	
**Sample size**	**Patients, ** ***n***	32,388	24,278	
	**Hospitals, ** ***n***	44	59	
**Patient characteristics**	**Age (median, IQR)**	76 (65–84)	78 (67–85)	0.0001
	**Stroke type (percent)**			
	Ischaemic	89.6%	88.9%	0.018
	Haemorrhage	10.4%	11.1%	
	**Female (percent)**	49.7%	52.0%	
	**OCSP type (percent)**			
	TACI	12.1%	11.8%	<0.0001
	LACI	17.3%	15.1%	
	POCI	10.3%	8.7%	
	PACI	57.6%	60.3%	
	Other	2.8%	4.1%	
	**Hypoxia (percent)**	16.5%	21.6%	<0.0001
	**Consciousness (percent)**			
	Fully conscious	76.5%	75.1%	<0.0001
	Reduced	19.0%	19.5%	
	Unconscious	4.6%	6.4%	
	**Independent prior to stroke (percent)**	84.6%	81.9%	<0.0001
	**Weekend admission (percent)**	25.9%	25.2%	0.07
	**Onset of symptoms before arrival, minutes (median, IQR)**	423 (102–1,058)	420 (102–1,099)	0.009
**Organisational characteristics**	**24-h thrombolysis service (percent)**	88.7%	67.8%	<0.0001
	**SU beds (median, IQR)**	19.5 (12.0–27.5)	20 (10.0–26.0)	0.0001
	**Nurses/ten beds on weekdays (median, IQR)**	2.2 (1.7–3.3)	1.9 (1.6–2.5)	0.0001
	**Nurses/ten beds on weekend (median, IQR)**	2.1(1.5–3.3)	1.8(1.4–2.5)	0.0001
	**Healthcare assistants/ten beds beds on weekdays (median, IQR)**	1.5 (1.0–1.8)	1.8 (1.5–2.5)	0.0001
	**Healthcare assistants/ten beds on weekend (median, IQR)**	1.4 (1.0–1.8)	1.8 (1.4–2.5)	0.0001
**Care quality**	**Only in ICU, HDU, or SU in first 24 h (percent)**	85.7%	70.6%	<0.0001
	**Antiplatelet agents within 24 h (or contraindicated) (percent)**	92.5%	88.2%	<0.0001
	**Brain scan within 24 h (percent)**	92.6%	90.2%	<0.0001
**Outcome**	**Mortality (percent)**			
	7 d	5.6%	7.2%	<0.0001
	30 d	11.8%	14.9%	<0.0001
	90 d	17.1%	21.1%	<0.0001

IQR, interquartile range; LACI, lacunar syndrome; PACI, partial anterior circulation syndrome; POCI, posterior circulation syndrome; TACI, total anterior circulation syndrome.

**Table 2:** Characteristics of patients and hospitals in SUs with stroke specialist physician rounds <7 d/wk.

Category	Characteristic	Stroke Specialist Physician Rounds		
		<5 d/wk	5 d/wk	6 d/wk
**Sample size**	**Patients, ** ***n***	4,959	16,355	2,964
	**Hospitals, ** ***n***	10	42	7
**Patient characteristics**	**Age (median, IQR)**	78 (68–85)	78 (67–85)	78 (67–85)
	**Stroke type (percent)**			
	Ischaemic	88.4%	89.1%	88.9%
	Haemorrhage	11.6%	10.9%	11.1%
	**Female (percent)**	52.1%	51.8%	53.5%
	**OCSP type (percent)**			
	TACI	10.9%	11.8%	13.3%
	LACI	18.0%	14.1%	16.2%
	POCI	7.9%	8.8%	9.3%
	PACI	57.6%	61.4%	59.0%
	Other	5.6%	4.0%	2.2%
	**Hypoxia (percent)**	17.6%	24.1%	15.0%
	**Consciousness (percent)**			
	Fully conscious	74.8%	74.0%	73.4%
	Reduced	19.0%	19.6%	20.2%
	Unconscious	6.2%	6.5%	6.4%
	**Independent prior to stroke (percent)**	79.7%	80.3%	76.2%
	**Weekend admission (percent)**	25.6%	25.3%	24.2%
	**Onset of symptoms before arrival, minutes (median, IQR)**	589 (130–1,121)	368 (95–961)	359 (89–996)
**Organisational characteristics**	**24-h thrombolysis service (percent)**	64.2%	74.0%	68.2%
	**SU beds (median, IQR)**	23 (14–31)	20 (13–27)	20 (8–28)
	**Nurses/ten beds on weekdays (median, IQR)**	1.7 (1.3–1.9)	1.8 (1.6–2.5)	2.5 (2.1–2.5)
	**Nurses/ten beds on weekend (median, IQR)**	1.5 (1.3–1.9)	1.8 (1.3–2.5)	2.1 (1.9–2.1)
	**Healthcare assistants/ten beds on weekend (median, IQR)**	1.8 (1.7–2.1)	2.0 (1.6–2.5)	2.2 (1.3–2.5)
**Care quality**	**Only in ICU, HDU, or SU in first 24 h (percent)**	62.4%	72.5%	74.1%
	**Antiplatelet agents within 24 h (or contraindicated) (percent)**	92.4%	86.7%	89.2%
	**Brain scan within 24 h (percent)**	84.6%	91.7%	90.9%
**Outcome**	**Mortality (percent)**			
	7 d	6.4%	7.4%	7.2%
	30 d	14.6%	15.1%	14.7%
	90 d	20.8%	21.3%	21.0%

IQR, interquartile range; LACI, lacunar syndrome; PACI, partial anterior circulation syndrome; POCI, posterior circulation syndrome; TACI, total anterior circulation syndrome.

Cox proportional hazards models were fitted in order to estimate the hazard ratio (HR) of death within 30 d of admission. Standard errors were estimated using the robust sandwich estimator, with clustering by hospital. Absolute effect sizes were computed using previously described methods [18]. The proportional hazards assumption was examined using log–log plots. Model discrimination was assessed by the Harrell's C statistic and was found to be good (0.88 for the model including patient prognostic variables). Possible non-linear associations between nursing ratios and mortality were included in the model through the use of cubic regression splines, using previously described methods [19]. The resulting model coefficients are not in themselves directly interpretable; we therefore plotted estimated HRs and 95% confidence intervals for values of nurse/bed ratio. A similar method was used to examine the interaction between weekend admission and nursing levels, by fitting multivariable fractional polynomial models [20].

Model fitting took into account a number of potential sources of confounding: patient-level prognostic factors, other measures of clinical staffing, the characteristics of the SU that patients were admitted to, and measures of the quality of care that patients received. Patient-level prognostic variables were age, stroke type (ischaemic versus haemorrhagic stroke), independence in activities of daily living prior to stroke (specified as a pre-morbid modified Rankin score of 0–2), hypoxia in the first 24 h of admission (defined as SaO_2_<95% without supplemental oxygen), the lowest level of consciousness recorded in the first 24 h (categorized into three levels: “fully conscious”, “reduced consciousness”, and “unconscious”), arm weakness, leg weakness, hemianopia, and dysphasia. Stroke organisational variables included the total number of SU beds and the presence of a 24/7 on-site stroke thrombolysis service. Staffing variables included the ratio of unregistered healthcare assistants/ten beds working on the weekend, the ratio of registered nurses/ten beds working on weekends, and the presence of 7-d/wk physician ward rounds. Care quality variables included whether the patient was managed solely in an optimal setting (SU, intensive care unit [ICU], or high dependency unit [HDU]) during the first 24 h of admission, administration of antiplatelet therapy if indicated (patients were coded as compliant if antiplatelet therapy was not indicated), and performance of a brain scan (computed tomography scan or MRI) within 24 h of admission.

SUs (*n* = 7) were not included if they had submitted data on fewer than 20 patients per quarter, on the assumption that data from these facilities were most at risk of bias from selective reporting. A complete case sensitivity analysis was also carried out including data from these hospitals. An analysis restricted to the subgroup of patients admitted on a weekday who were discharged home or died before the weekend was also conducted. These patients have no direct exposure to weekend staffing levels, and so despite not being representative of the entire sample, an association between weekend staffing and mortality in this group might be an indicator of bias from unmeasured confounding.

Categorical variables were compared using the Pearson Chi-squared test, and continuous variables using the Kruskall-Wallis test, using a significance level of 0.01. Analysis was carried out using Stata 12.0. The Stata modules mfpi and mvrs were used to fit the fractional polynomial and cubic regression spline models.

## Results

Of 56,666 stroke patients admitted to the 103 hospitals included in this sample, 14,475 (25.6%) were admitted on a weekend. Crude mortality by 30 d was higher for patients admitted on a weekend (HR 1.16, 1.09–1.24), but weekend admission was of only borderline significance after adjusting for patient case mix (adjusted HR 1.05, 1.00–1.11). However, this overall picture masked heterogeneity between SUs in their organisational characteristics, patient demographics, and outcomes.

Almost half (44/103) of SUs provided physician ward rounds 7 d/wk (Table 1), and the great majority (42/59) of the remainder had rounds 5 d of the week (Monday–Friday) (Table 2). There were significant differences in the patient demographics between SUs: patients admitted to SUs with rounds 7 d/wk were younger, more likely to have lacunar or posterior circulation stroke syndromes, and less likely to be hypoxic, unconscious, or dependent in activities of daily living prior to stroke (Table 1). Absolute differences in patient characteristics were overall quite modest—the large sample size meant that significance tests were sensitive to small differences. SUs with rounds 7 d/wk also had higher nurse/bed ratios and were more likely to have a 24/7 on-site thrombolysis service (Table 1). Patients admitted to these SUs were more likely to receive all three of the care quality measures (Table 1). Crude mortality was lower at 7 d, 30 d, and 90 d after admission to a SU with ward rounds 7 d/wk (mortality at 30 d, 11.8% versus 14.9%, *p*<0.0001).

After adjusting for differences in case mix, organisational variables, nurse staffing, and care quality variables, there was no evidence of a significant relationship between SUs having rounds by stroke specialist physicians 7 d/wk and mortality (Table 3).

**Table 3:** Hazard ratios of death by 30 d in univariable and multivariable models.

Model	*N*	Stroke Specialist Physician Rounds			
		7 d/wk		<7 d/wk	
		Weekday Admission	Weekend Admission	Weekday Admission	Weekend Admission
Univariable	56,211	Reference	1.30 (1.17–1.46)	1.18 (1.08–1.30)	1.49 (1.32–1.69)
Adjusted for patient case mix, organisational characteristics, staffing, and care quality	56,211	Reference	0.96 (0.85–1.10)	1.05 (0.97–1.14)	1.04 (0.91–1.18)

Patient case mix: age, sex, stroke type, consciousness level, hypoxia, independence in activities of daily living before stroke, arm weakness, leg weakness, dysphasia, hemianopia. Organisational characteristics: total number of SU beds, 24/7 on-site stroke thrombolysis service. Staffing: average number of registered nurses/ten beds on weekdays, average number of registered nurses/ten beds on weekends, average number of healthcare assistants/ten beds on weekends. Care quality: only in ICU, HDU, or SU in first 24 h, antiplatelet therapy within 24 h (if indicated), brain scan within 24 h of admission.

**Figure 1:**
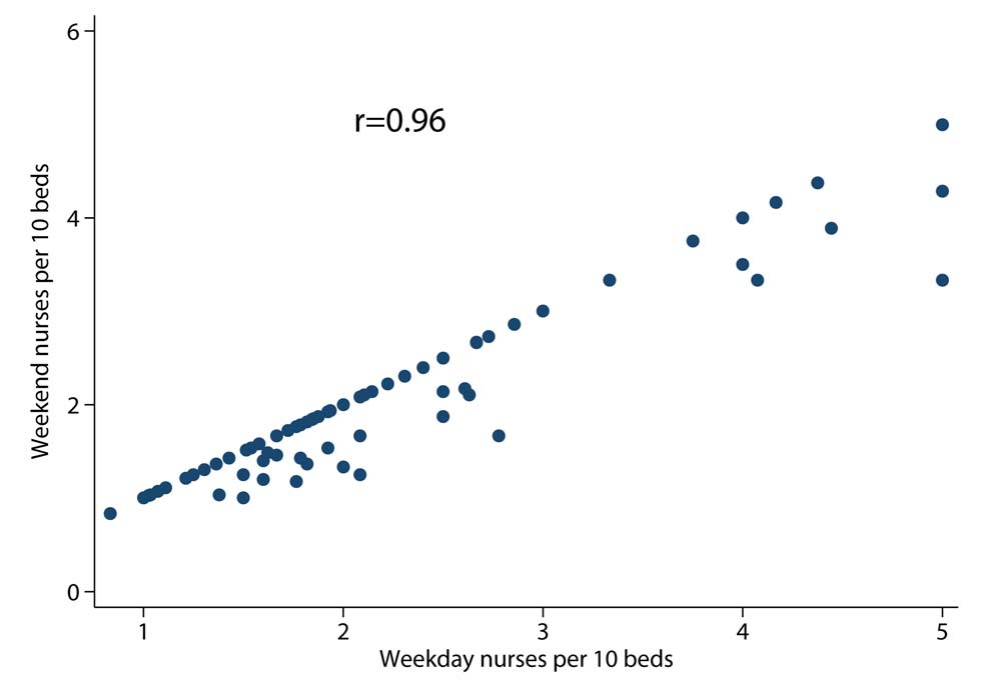
Scatter plot of weekday nurses per ten beds versus weekend nurses per ten beds.

There was 5-fold variation between SUs in the nurse/bed ratio on weekends, and the nurse/bed ratio on weekends was highly correlated (Pearson *r* = 0.96) with the nurse/bed ratio on weekdays (Figure 1). Again, there was evidence of significant differences in important patient prognostic variables between SUs (age, OCSP type, hypoxia, consciousness, and pre-stroke independence) but no clear linear trends between the nursing ratios and case mix variables (Table 4). SUs with higher nurse/bed ratios had fewer beds and higher nurse/bed ratios during the week and were more likely to also have physician rounds 7 d/wk (Table 4). Care quality was highest, and crude mortality lowest, in SUs with ratios of ≥3.0 nurses/ten beds compared to SUs with lower nurse/bed ratios.

**Table 4:** Characteristics of patient population, organisational characteristics of stroke units, process measures of care quality, and crude mortality rate by weekend nurse/bed ratio.

Category	Characteristic	Nursing Ratios on the Weekend (Nurses/Ten Beds)				*p*-Value
		≥3.0	2.0–2.9	1.5–1.9	<1.5	
**Sample size**	**Patients, ** ***n***	17,922	12,253	12,267	13,323	
	**Hospitals, ** ***n***	21	26	27	26	
**Patient characteristics**	**Age (median, IQR)**	76 (66–84)	77 (67–85)	77 (66–84)	78 (67–85)	<0.0001
	**Stroke type (percent)**					0.06
	Ischaemic	89.7%	88.8%	89.3%	89.3%	
	Haemorrhage	10.3%	11.2%	10.7%	10.7%	
	**Female (percent)**	49.7%	51.6%	50.6%	51.4%	
	**OCSP type (percent)**					<0.0001
	TACI	10.3%	14.9%	12.8%	11.1%	
	LACI	17.0%	15.8%	18.4%	14.0%	
	POCI	10.3%	9.4%	10.6%	7.9%	
	PACI	59.3%	56.9%	55.1%	62.7%	
	Other	3.1%	3.0%	3.1%	4.3%	
	**Hypoxia (percent)**	15.7%	18.7%	17.0%	24.1%	<0.0001
	**Consciousness (percent)**					<0.0001
	Fully conscious	76.9%	72.9%	76.2%	75.7%	
	Reduced	19.5%	20.5%	18.0%	18.3%	
	Unconscious	3.6%	6.6%	5.8%	6.0%	
	**Independent prior to stroke (percent)**	79.7%	79.3%	80.3%	80.7%	0.001
	**Weekend admission (percent)**	25.5%	26.4%	24.8%	25.8%	0.6
	**Onset of symptoms before arrival, minutes (median, IQR)**	480 (110–1,201)	358 (95–975)	413 (100–1,030)	490 (104–1,063)	0.0001
**Organisational characteristics**	**24-h thrombolysis service (percent)**	90.4%	76.9%	66.7%	76.9%	<0.0001
	**SU beds (median, IQR)**	10 (4–16)	18 (8–23)	26 (19–31)	24 (20–28)	<0.0001
	**Nurses/ten beds on weekdays**	4.2 (3.3–5.0)	2.5(2.2–2.6)	1.7(1.7–1.9)	1.4 (1.2–1.6)	<0.0001
	**Physician ward rounds 7 d/wk (percent)**	87.5%	63.1%	54.5%	44.8%	<0.0001
	**Healthcare assistants/ten beds on weekdays**	1.7 (1.1–3.1)	1.8 (1.1–2.5)	1.6 (1.3–1.7)	1.7 (1.4–2.1)	0.66
	**Healthcare assistants/ten beds on weekend**	1.7 (1.1–3.1)	1.8 (1.1–2.5)	1.6(1.3–1.7)	1.7 (1.4–2.1)	0.66
**Care quality**	**Only in ICU, HDU, or SU in first 24 h (percent)**	88.2%	80.8%	76.0%	69.2%	<0.0001
	**Antiplatelet agents within 24 h (if indicated) (percent)**	92.2%	92.4%	90.8%	87.0%	<0.0001
	**Brain scan within 24 h (percent)**	92.9%	92.2%	90.6%	90.3%	<0.0001
**Outcomes**	**Mortality (percent)**					
	7 d	4.5%	7.5%	6.6%	7.1%	<0.0001
	30 d	10.2%	14.7%	13.8%	14.6%	<0.0001
	90 d	15.5%	20.8%	19.7%	20.3%	<0.0001

IQR, interquartile range; LACI, lacunar syndrome; PACI, partial anterior circulation syndrome; POCI, posterior circulation syndrome; TACI, total anterior circulation syndrome.

**Figure 2:**
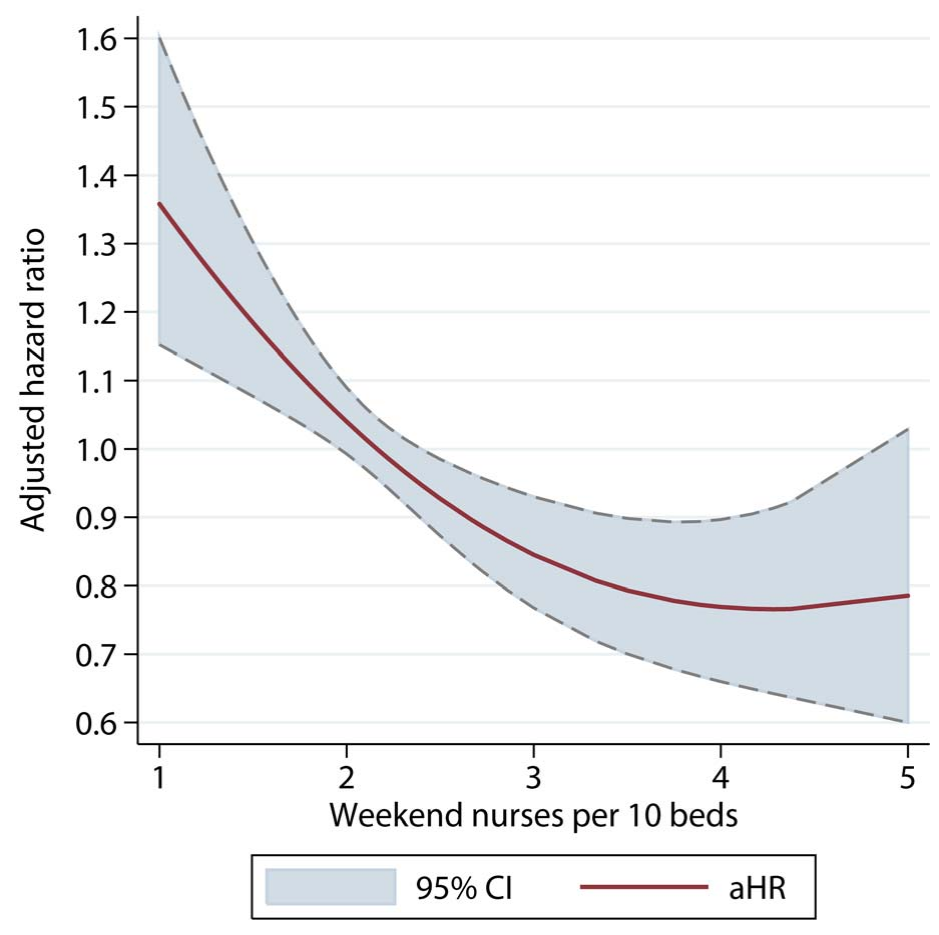
Adjusted hazard ratio of 30-d mortality of patients admitted on weekends, by ratio of registered nurses per ten beds on the weekend. HRs adjusted for patient case mix, organisational characteristics, staffing, and care quality.

**Figure 3:**
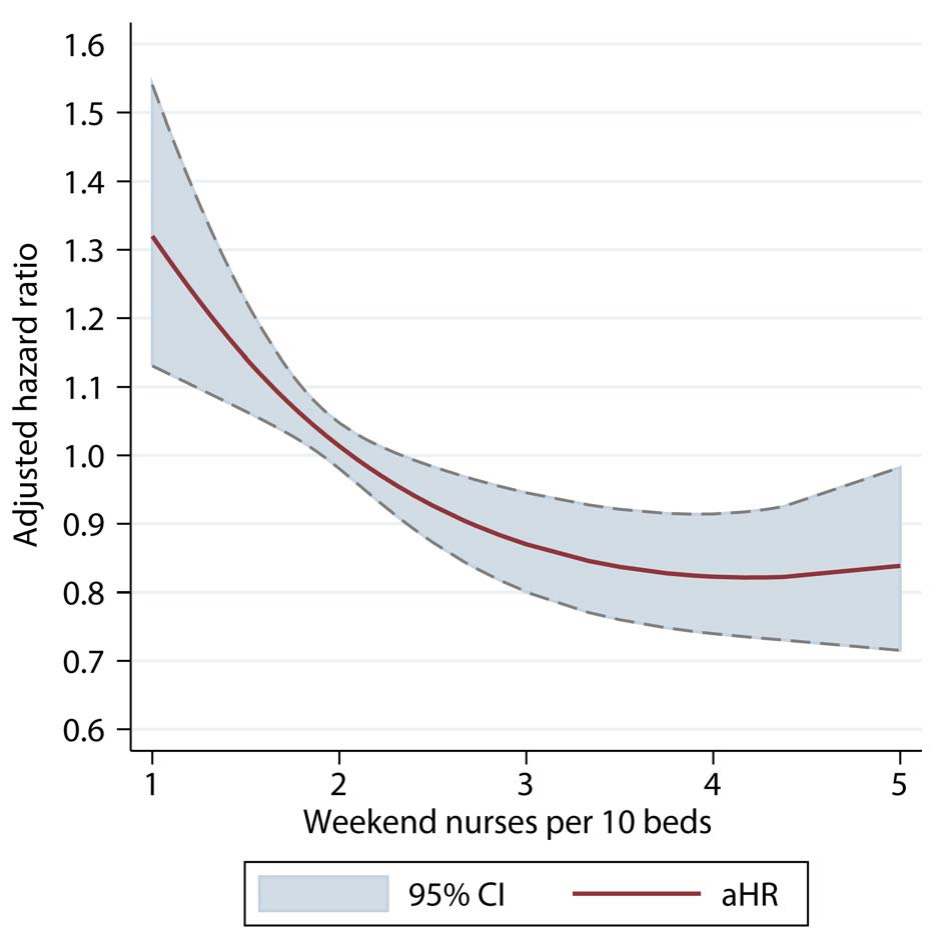
Adjusted hazard ratio of 30-d mortality of patients admitted on weekdays, by ratio of registered nurses per ten beds on the weekend. HRs adjusted for patient case mix, organisational characteristics, staffing, and care quality.

There was a dose–response relationship between weekend nurse/bed ratios and the estimated risk of death for patients admitted on the weekends in both univariable and multivariable analyses. In multivariable analysis adjusting for patient case mix, organisational characteristics, physician and healthcare assistant staffing, and care quality, higher nursing ratios were associated with significantly reduced risk of death, and lower nursing ratios with higher risk of death (Figure 2). A below-average nurse/bed ratio was associated with as much as a 35% increased risk of death, with the highest risk associated with the lowest nurse/bed ratios. By contrast, above-average nurse/bed ratios were associated with a reduced risk of death of 20%–30% (Figure 2). A very similar association was observed for patients admitted on weekdays: high weekend nurse/bed ratios were associated with reduced mortality and vice versa, with a similar estimated effect size as for weekend admissions (Figure 3). The relationship between mortality and nurse/bed ratios was similar in the secondary analysis using categorical rather than continuous nurse/bed ratio (Table 5).

**Table 5:** Hazard ratios of death by 30 d in univariable and multivariable models.

Model	*n*	Nursing Ratios on the Weekend (Nurses/Ten Beds)							
		≥3		2.0–2.9		1.5–1.9		<1.5	
		Weekday	Weekend	Weekday	Weekend	Weekday	Weekend	Weekday	Weekend
Univariable	56,211	Reference	1.00 (0.90–1.12)	1.41 (1.21–1.62)	1.70 (1.43–2.01)	1.31 (1.12–1.55)	1.63 (1.34–1.97)	1.39 (1.17–1.65)	1.70 (1.44–2.00)
Adjusted for patient case mix, organisational characteristics, staffing, and care quality	56,211	Reference	0.93 (0.83–1.05)	1.11 (0.96–1.29)	1.27 (1.08–1.49)	1,22 (1.07–1.39)	1.29 (1.11–1.50)	1.31 (1.11–1.53)	1.48 (1.25–1.75)
Adjusted for patient case mix, organisational characteristics, staffing, and care quality—only patients with no exposure to the weekend	9,396	Reference	—	1.22 (0.95–1.57)	—	1.12 (0.90–1.38)	—	1.14 (0.90–1.45)	—

Patient case mix: age, sex, stroke type, consciousness level, hypoxia, independence in activities of daily living before stroke, arm weakness, leg weakness, dysphasia, hemianopia. Organisational characteristics: total number of SU beds, 24/7 on-site stroke thrombolysis service. Staffing: physician ward rounds 7 d/wk, number of healthcare assistants/ten beds on weekends. Care quality: only in ICU, HDU, or SU in first 24 h, antiplatelet therapy within 24 h (if indicated), brain scan within 24 h of admission.

**Figure 4:**
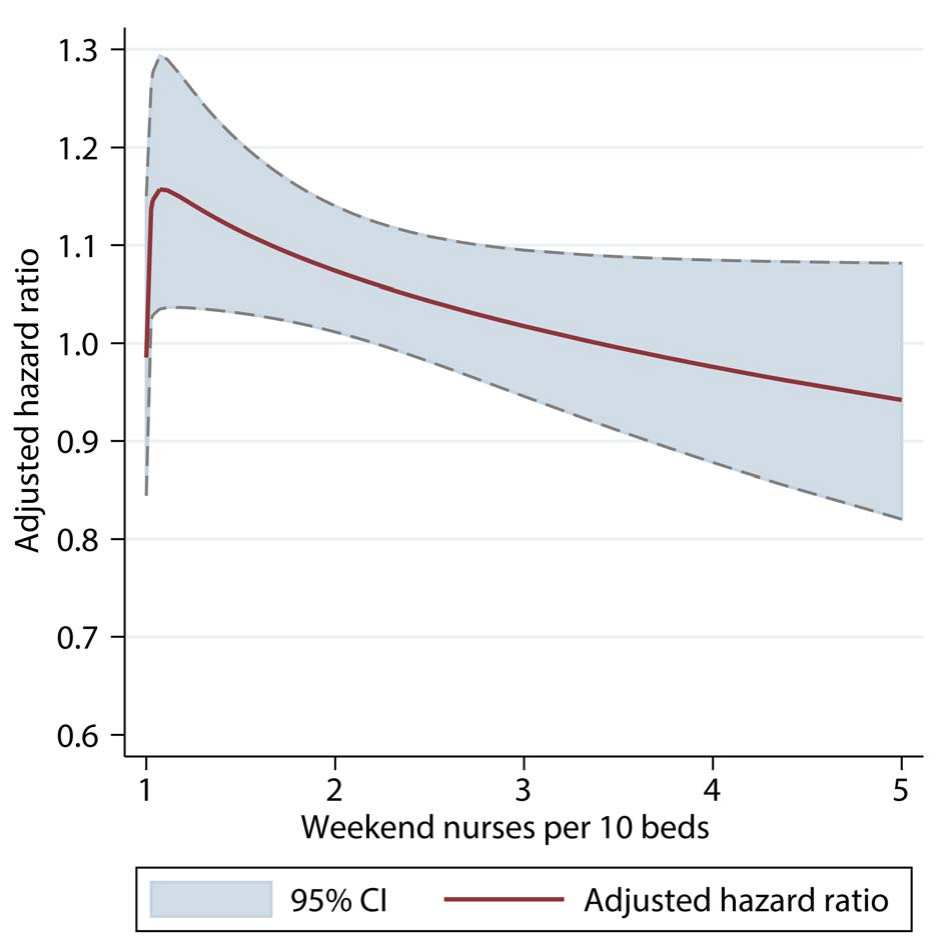
Interaction between nursing ratios and weekend admission. The adjusted HR is the difference in hazard associated with admission on a weekend versus a weekday, by ratio of registered nurses per ten beds on a weekend. HRs adjusted for patient case mix, organisational characteristics, staffing, and care quality.

There was also evidence of an interaction between weekend admission and risk of mortality. Patients admitted on a weekend to a SU with below-average nurse/bed ratios had a higher risk of death than those admitted on a weekday (adjusted HR 1.11, 95% CI 1.03–1.20, for 1.5 nurses/ten beds); by contrast, weekend admission to a SU with above-average nurse/bed ratios was not associated with an excess of mortality risk compared to patients admitted during a weekday (Figure 4).

If the observed associations were causal, the adjusted absolute difference in estimated 30-d mortality for admission to a SU with a ratio of 1.5 nurses/ten beds (15.2%, 95% CI 13.8%–16.4%) versus 3.0 nurses/ten beds (11.2%, 95% 10.2%–12.1%) on a weekend was 4.0% (95% CI 1.7%–6.3%). The number needed to harm through admission to a SU with 1.5 nurses/ten beds compared to 3.0 nurses/ten beds was 25 (95% CI 16–59).

**Figure 5:**
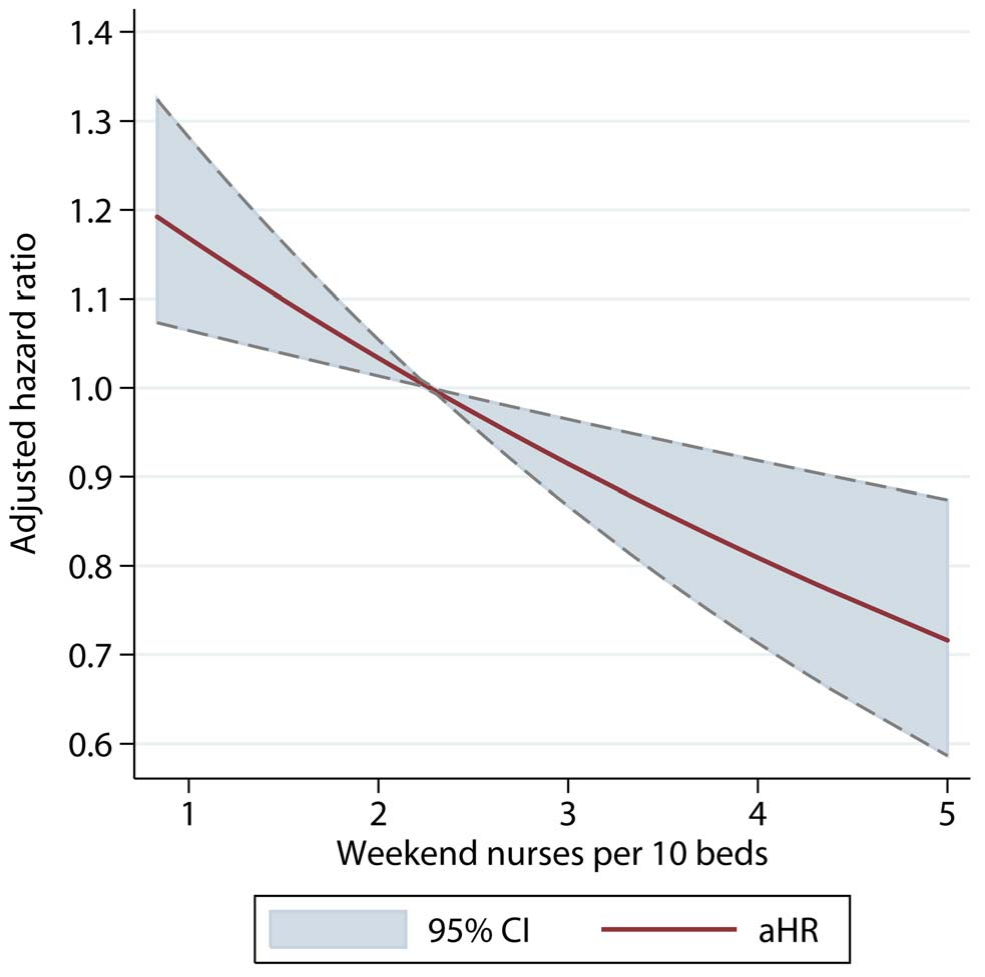
Adjusted hazard ratio of 30-d mortality of patients admitted on weekends, by ratio of registered nurses per ten beds on the weekend. Complete case analysis using data from all hospitals in SINAP, irrespective of case ascertainment.

**Figure 6:**
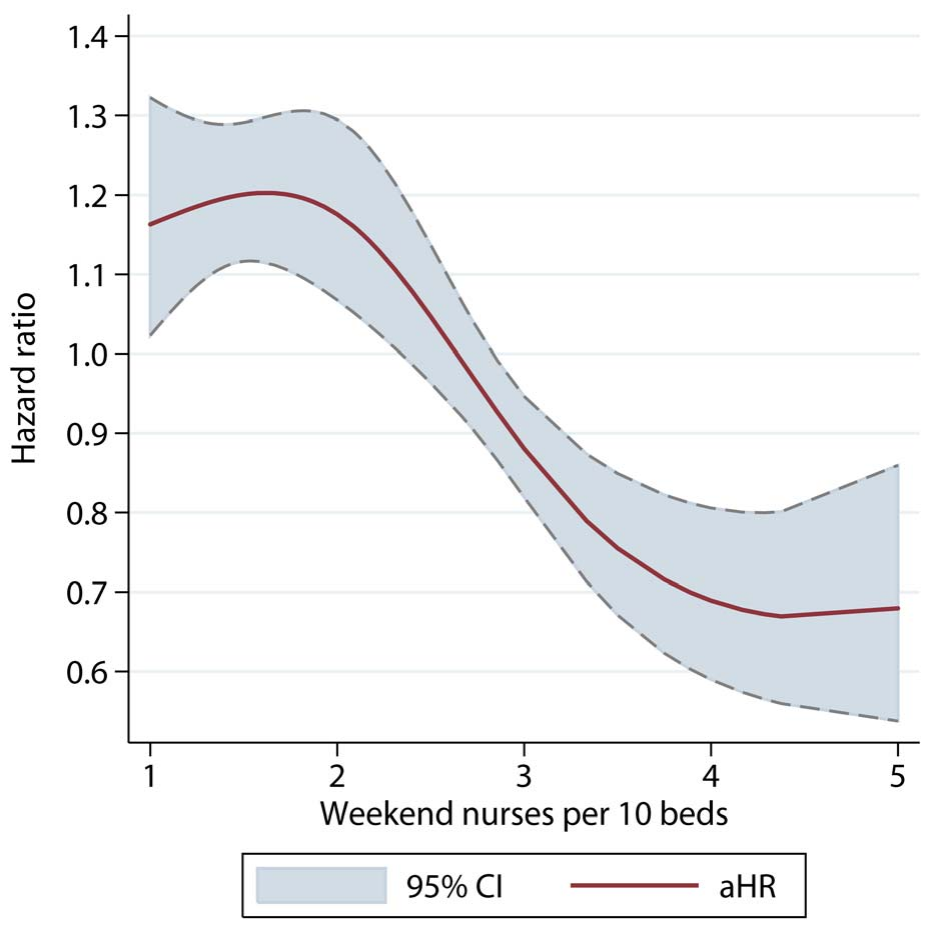
Unadjusted hazard ratio of 30-d mortality of patients admitted on weekends, by ratio of registered nurses per ten beds on the weekend.

In the sensitivity analysis of the subgroup of the 9,535 patients with no direct exposure to weekend staffing levels, there was no significant association between estimated mortality risk and nurse/bed ratios (Table 5), although the smaller sample size could have resulted in a type II error. The complete case analysis (Figure 5) and unadjusted analyses (Figure 6) yielded very similar results to the main analysis.

## Discussion

In this large, “real world” multicentre sample of SUs in England, there was overall only a weak association between weekend admission and mortality, but this masked important variation between SUs in their organisation of weekend care and mortality outcomes. There was wide variation in the intensity of clinical staffing on weekends, and evidence that a significant proportion of SUs were already providing weekend staffing levels similar to those during the week. After adjusting for patient-level and organisational variables, there was no evidence that having stroke specialist physician ward rounds 7 d/wk (as opposed to fewer than 7 d/wk) independently influenced an association between weekend admission and mortality. By contrast, there was evidence of a dose–response relationship between mortality and nursing ratios on the weekend (with lower nurse/bed ratios being associated with higher mortality risk), which was consistent in a variety of models adjusting for other potential confounders. These observational data support the hypothesis that nursing levels are an important mediator of excess mortality for patients admitted with stroke on a weekend.

Numerous observational studies have reported an association between weekend admission for stroke and higher mortality, in both specialized stroke care units [21]–[23] and a variety of other settings [2]–[6]. However, the association has not been found in all studies [24], and the question about whether, and if so, how, different models of care deliver equivalent outcomes to patients admitted on weekends has received little research attention. Reduced levels of staffing on the weekend have been proposed as one possible cause of excess mortality [25],[26]. There is, however, very little evidence that weekend staffing levels are associated with patient outcomes, and we are aware of no studies specifically addressing this question in stroke care. The strongest evidence concerns staffing in ICUs, where there is evidence that daily rounds by multidisciplinary teams reduce mortality [27]. However, despite a variety of observational data suggesting that more intensive ICU specialist physician staffing overnight is associated with better outcomes [28],[29], this hypothesis was not supported in a recent randomised controlled trial [30]. Previous data from England demonstrated lower weekend mortality in hospitals with higher levels of daily physician presence for acute medical admissions, although no effect sizes were reported, making the results difficult to interpret [31]. The evidence concerning nursing ratios is stronger, although again, there are no specific studies of this in stroke care. Studies in a variety of settings have generally demonstrated lower mortality with higher levels of nurse staffing [32]–[36], but have not specifically studied nurse staffing on weekends.

Our study shows that most of the SUs studied had physician rounds 5 or 7 d per week. The lack of an association of mortality with daily physician ward rounds might be explained by the observation that the majority of units not providing rounds 7 d/wk instead had rounds 5 d/wk: the difference in patients' exposure to the frequency of physician rounds was therefore small. By contrast, weekend nursing ratios were strongly associated with mortality outcomes, not only for patients admitted on a weekend but also for those admitted on a weekday. The latter finding may be a consequence of the high degree of correlation between weekday and weekend nurse/bed ratios and may reflect a general association between nursing levels and mortality risk. However, it is also possible that low nurse levels on weekends may incur risk to all inpatients, not just new admissions. Although these are observational data, the dose–response relationship is suggestive of a causal relationship between nursing ratios and mortality. The mechanism for this is unclear, but there is evidence that basic care needs are more likely to be missed when nurses are busy [37], and higher nurse ratios might prevent mortality through quicker recognition and management of deterioration, prevention of stroke complications [38], and allowing staff more time to attend to basic nutrition and hydration. The finding of an interaction between weekend admission and nurse staffing might explain why some studies have not observed excess weekend mortality—the “weekend effect” might occur only in healthcare settings where nurse staffing levels are low.

This study used a large, nationally representative sample and has the advantage of making use of detailed clinical data (with high levels of completeness) rather than administrative datasets. The stroke services sampled were all part of the National Health Service, which provides universal healthcare coverage to all residents of the United Kingdom, funded through general taxation and largely free at the point of use. The results are therefore unlikely to be biased by differences in funding between hospitals or in patients' ability to access care because of income or insurance status. There are, however, several limitations to this study. First, the study used an ecological design and assumed that the staffing levels were representative of the care (e.g., nursing contact time or review on a weekend round) that patients actually received. In addition, although we have attempted to control for a number of sources of confounding, it is not possible to exclude confounding from unmeasured patient or SU characteristics. There are many variables that are potentially important but were not available in the datasets, such as measures of socioeconomic status. Mortality, although important, is clearly not the only outcome of interest after stroke, and no data were available on other relevant outcomes such as disability or patient experience. Data were also collected on a voluntary basis by hospitals, and although we allowed for this in the analysis, we cannot exclude the possibility of bias from selective or inaccurate reporting. Not all SUs in England participated, and the results may not be representative of non-participating sites or of stroke care outside England. Many of these limitations are inherent to observational data and highlight the importance of controlled evaluations of staffing levels, through, for example, cluster randomised controlled trials or interrupted time series studies.

This study is one of the first in any healthcare setting to specifically examine the relationship between the organisation of care on weekends and mortality. Despite the fact that staff account for the great majority of healthcare spending (64% in the National Health Service) [39], there remains very little research into the effect of clinical staffing levels on patient outcomes. Controlled studies of different models of physician and nursing staffing seem both feasible and important, given the potentially large impact on patient outcomes and the high costs to health systems of increasing staffing levels on weekends. In the meantime, these data support the provision of higher weekend registered nurse/bed ratios in SUs.

## Supporting Information

Checklist S1 **STROBE checklist.**
(DOC)

## Abbreviations

aHR: adjusted hazard ratio
HDU: high dependency unit
HR: hazard ratio
ICU: intensive care unit
OCSP: Oxfordshire Community Stroke Project
SINAP: Stroke Improvement National Audit Programme
SU: stroke unit

## References

[1] National Health Service Commissioning Board (2012) Everyone counts: planning for patients 2013/14. Leeds: National Health Service England. Available: http://www.england.nhs.uk/wp-content/uploads/2012/12/everyonecounts-planning.pdf. Accessed 31 July 2013.

[2] Kostis WJ, Demissie K, Marcella SW, Shao YH, Wilson AC, et al (2007) Weekend versus weekday admission and mortality from myocardial infarction. N Engl J Med 356: 1099–1109. 17360988 10.1056/NEJMoa063355

[3] Bell CM, Redelmeier DA (2001) Mortality among patients admitted to hospitals on weekends as compared with weekdays. N Engl J Med 345: 663–668. 11547721 10.1056/NEJMsa003376

[4] Cram P, Hillis SL, Barnett J, Rosenthal GE (2004) Effects of weekend admission and hospital teaching status on in-hospital mortality. Am J Med 117: 151–157. 15276592 10.1016/j.amjmed.2004.02.035

[5] Aylin P, Yunus A, Bottle A, Majeed A, Bell D (2010) Weekend mortality for emergency admissions. A large multicentre study. Qual Saf Health Care 19: 213–217. 20110288 10.1136/qshc.2008.028639

[6] Mohammed MA, Sidhu KS, Rudge G, Stevens AJ (2012) Weekend admission to hospital has a higher risk of death in the elective setting than in the emergency setting: a retrospective database study of national health service hospitals in England. BMC Health Serv Res 12: 87. 22471933 10.1186/1472-6963-12-87 PMC3341193

[7] Handel AE, Patel SV, Skingsley A, Bramley K, Sobieski R, et al (2012) Weekend admissions as an independent predictor of mortality: an analysis of Scottish hospital admissions. BMJ Open 2: e001789. 10.1136/bmjopen-2012-001789 PMC3533021 23135542

[8] Graham MM, Ghali WA, Southern DA, Traboulsi M, Knudtson ML, et al (2011) Outcomes of after-hours versus regular working hours primary percutaneous coronary intervention for acute myocardial infarction. BMJ Qual Saf 20: 60–67. 10.1136/bmjqs.2010.041137 PMC3022364 21228077

[9] Rathod KS, Jones DA, Gallagher SM, Bromage DI, Whitbread M, et al (2013) Out-of-hours primary percutaneous coronary intervention for ST-elevation myocardial infarction is not associated with excess mortality: a study of 3347 patients treated in an integrated cardiac network. BMJ Open 3: e003063. 10.1136/bmjopen-2013-003063 PMC3696864 23811175

[10] Lozano R, Naghavi M, Foreman K, Lim S, Shibuya K, et al (2012) Global and regional mortality from 235 causes of death for 20 age groups in 1990 and 2010: a systematic analysis for the Global Burden of Disease Study 2010. Lancet 380: 2095–2128. 23245604 10.1016/S0140-6736(12)61728-0 PMC10790329

[11] Murray CJ, Vos T, Lozano R, Naghavi M, Flaxman AD, et al (2012) Disability-adjusted life years (DALYs) for 291 diseases and injuries in 21 regions, 1990–2010: a systematic analysis for the Global Burden of Disease Study 2010. Lancet 380: 2197–2223. 23245608 10.1016/S0140-6736(12)61689-4

[12] Saka O, McGuire A, Wolfe C (2009) Cost of stroke in the United Kingdom. Age Ageing 38: 27–32. 19141506 10.1093/ageing/afn281

[13] (2014) SINAP (Stroke Improvement National Audit Programme). London: Royal College of Physicians. Available: http://www.rcplondon.ac.uk/sinap. Accessed 31 July 2013.

[14] Bamford J, Sandercock P, Dennis M, Burn J, Warlow C (1991) Classification and natural history of clinically identifiable subtypes of cerebral infarction. Lancet 337: 1521–1526. 1675378 10.1016/0140-6736(91)93206-o

[15] Office for National Statistics (2014) Death registrations. Available: http://www.ons.gov.uk/ons/taxonomy/index.html?nscl=Death+Registrations. Accessed 21 July 2014.

[16] Health and Social Care Information Centre (2014) Mortality data from the Office for National Statistics. Available: http http://www.hscic.gov.uk/onsmortality. Accessed 21 July 2014.

[17] (2014) SSNAP Acute Organisational Audit. London: Royal College of Physicians. Available: http://www.rcplondon.ac.uk/projects/ssnap-acute-organisational-audit. Accessed 31 July 2013.

[18] Altman DG, Andersen PK (1999) Calculating the number needed to treat for trials where the outcome is time to an event. BMJ 319: 1492–1495. 10582940 10.1136/bmj.319.7223.1492 PMC1117211

[19] Royston P, Sauerbrei W (2007) Multivariable modelling with cubic regression splines: a principled approach. Stata J 7: 45–70.

[20] Royston P, Sauerbrei W (2004) A new approach to modelling interactions between treatment and continuous covariates in clinical trials using fractional polynomials. Stat Med 23: 2509–2525. 15287081 10.1002/sim.1815

[21] Ovbiagele BV (2010) Nationwide trends in in-hospital mortality among patients with stroke. Stroke 41: 1748–1754. 20558829 10.1161/STROKEAHA.110.585455

[22] Fang J, Saposnik G, Silver FL, Kapral MK (2010) Investigators of the Registry of the Canadian Stroke Network (2010) Association between weekend hospital presentation and fatality. Neurology 75: 1589–1596. 21041782 10.1212/WNL.0b013e3181fb84bc

[23] Reeves MJ, Smith E, Fonarow G, Hernandez A, Pan W, et al (2009) Off-hour admission and in-hospital stroke case fatality in the get with the guidelines-stroke program. Stroke 40: 569–576. 18988914 10.1161/STROKEAHA.108.519355

[24] Albright KC, Raman R, Ernstrom K, Hallevi H, Martin-Schild S, et al (2009) Can comprehensive stroke centers erase the ‘weekend effect’? Cerebrovasc Dis 27: 107–113. 19039213 10.1159/000177916 PMC2790057

[25] Goddard AF, Lees P (2012) Higher senior staffing levels at weekends and reduced mortality. BMJ 344: e367. 22234913 10.1136/bmj.e67

[26] Mourad M, Adler J (2011) Safe, high quality care around the clock: what will it take to get us there? J Gen Intern Med 26: 948–950. 21751054 10.1007/s11606-011-1795-5 PMC3157519

[27] Kim MM, Barnato AE, Angus DC, Fisher LF, Kahn JM (2010) The effect of multidisciplinary care teams on intensive care mortality. Arch Intern Med 170: 369–376. 20177041 10.1001/archinternmed.2009.521 PMC4151479

[28] Wallace DJ, Angus DC, Barnato AE, Kramer AA, Kahn JM (2012) Nighttime intensivist staffing and mortality among critically ill patients. N Engl J Med 366: 2093–2101. 22612639 10.1056/NEJMsa1201918 PMC3979289

[29] Pronovost PJ, Angus DC, Dorman T, Robinson KA, Dremisov TT, et al (2002) Physician staffing patterns and clinical outcomes in critically ill patients. JAMA 288: 2151–2162. 12413375 10.1001/jama.288.17.2151

[30] Kerlin MP, Small DS, Cooney E, Fuchs BD, Bellini LM, et al (2013) A randomized trial of nighttime physician staffing in an intensive care unit. N Engl J Med 368: 2201–2209. 23688301 10.1056/NEJMoa1302854 PMC3732473

[31] Bell D, Lambourne A, Percival F, Laverty AA, Ward DK (2013) Consultant input in acute medical admissions and patient outcomes in hospitals in England: a multivariate analysis. PLoS ONE 8: e61476. 23613858 10.1371/journal.pone.0061476 PMC3629209

[32] Schubert M, Clarke SP, Aiken LH, de Geest S (2012) Associations between rationing of nursing care and inpatient mortality in Swiss hospitals. Int J Qual Health Care 24: 230–238. 22457240 10.1093/intqhc/mzs009

[33] Profit J, Petersen LA, McCormick MC, Escobar GJ, Colman-Phox K, et al (2010) Patient-to-nurse ratios and outcomes of moderately preterm infants. Peditarics 125: 320–326. 10.1542/peds.2008-3140 PMC3151172 20064868

[34] Kane RL, Shamliyan T, Mueller C, Duval S, Wilt TJ (2007) Nurse staffing and quality of patient care. Rockville (Maryland): Agency for Healthcare Research and Quality. Available: http://archive.ahrq.gov/downloads/pub/evidence/pdf/nursestaff/nursestaff.pdf. Accessed 31 July 2013.

[35] Butler M, Collins R, Halligan P, O'Mathuna DP, Schultz TJ, et al (2011) Hospital nurse staffing models and patient and staff related outcomes. Cochrane Database Syst Rev 2011: CD007019. 21735407 10.1002/14651858.CD007019.pub2

[36] Needleman J, Buerhaus P, Pankratz VS, Leibson CL, Stevens SR, et al (2011) Nurse staffing and inpatient hospital mortality. N Engl J Med 364: 1037–1045. 21410372 10.1056/NEJMsa1001025

[37] Ball JE, Murrells T, Rafferty AM, Morrow E, Griffiths P (2013) “Care left undone” during nursing shifts: associations with workload and perceived quality of care. BMJ Qual Saf 23: 116–125 doi: 10.1136/bmjqs-2012-001767 10.1136/bmjqs-2012-001767 PMC3913111 23898215

[38] Govan L, Langhorne P, Weir CJ (2007) Does the prevention of complications explain the survival benefit of organized inpatient (stroke unit) care?: A further analysis of a systematic review. Stroke 38: 2536–2540. 17690313 10.1161/STROKEAHA.106.478842

[39] Charlesworth A (2013) The anatomy of health spending 2011/12: a review of NHS expenditure and labour productivity. London: Nuffield Trust. Available: http://www.nuffieldtrust.org.uk/publications/anatomy-health-spending-201112-review-nhs-expenditure-and-labour-productivity. Accessed 31 July 2013.

